# New antiarrhythmic targets to control intracellular calcium handling

**DOI:** 10.1007/s12471-014-0549-5

**Published:** 2014-04-15

**Authors:** H. E. Driessen, V. J. A. Bourgonje, T. A. B. van Veen, M. A. Vos

**Affiliations:** Department of Medical Physiology, Division of Heart & Lungs, University Medical Center Utrecht, Yalelaan 50, 3584 CM Utrecht, the Netherlands

**Keywords:** Arrhythmia, Calcium, Drugs, Treatment

## Abstract

Sudden cardiac death due to ventricular arrhythmias is a major problem. Drug therapies to prevent SCD do not provide satisfying results, leading to the demand for new antiarrhythmic strategies. New targets include Ca^2+^/Calmodulin-dependent protein kinase II (CaMKII), the Na/Ca exchanger (NCX), the Ryanodine receptor (RyR, and its associated protein FKBP12.6 (Calstabin)) and the late component of the sodium current (*I*_*Na-Late*_), all related to intracellular calcium (Ca^2+^) handling. In this review, drugs interfering with these targets (SEA-0400, K201, KN-93, W7, ranolazine, sophocarpine, and GS-967) are evaluated and their future as clinical compounds is considered. These new targets prove to be interesting; however more insight into long-term drug effects is necessary before clinical applicability becomes reality.

## Introduction

Sudden cardiac death (SCD) due to arrhythmias is a major problem in the Western population [1]. SCD occurs when, due to a trigger, sinus rhythm lapses into ventricular tachycardia (VT) which can deteriorate into ventricular fibrillation. At that stage of rhythm disorder, the contractile performance of the heart is severely compromised and eventually results in asystole and, as such, arrest of effective circulation [2]. Conditions such as congenital heart disease, cardiomyopathies and risk factors (e.g. smoking or hypertension) can be the underlying mechanisms of SCD [2]. In patients with dilated cardiomyopathy 30 % will suffer from SCD [3]. SCD in patients with end-stage heart failure is mostly due to mechanical dysfunction based on structural and contractile changes [3]. Prevention strategies focus on antiarrhythmic drugs and implantation of a cardiac defibrillator. Current drug therapies, though effective, also have major drawbacks (as discussed below) and the results have partially been disappointing [4].

Besides the classical modulation of sarcolemmal ion channels by drugs (including both activation and block), new antiarrhythmic targets emerge that can be of great interest in pharmacological treatment of VT in cardiac disease. Especially arrhythmias that occur in hypertrophy and heart failure have our attention, because of the difficulty in treating these patients effectively. Besides disappointing data concerning antiarrhythmic efficacy, current therapies such as blockade of the late-type calcium current (*I*_*Ca-L*_), are known to affect haemodynamics negatively.

These new targets include Ca^2+^/Calmodulin-dependent protein kinase II (CaMKII), the Na/Ca exchanger (NCX), the Ryanodine receptor (RyR, and its associated protein FKBP12.6) and the late component of the sodium current (I_Na-Late_), all related to intracellular calcium (Ca^2+^) handling of the cardiac myocyte. With this development, emphasis of antiarrhythmic drugs seems to shift therapy more in the direction of focal arrhythmias, which can be caused by delayed (DAD) or early after depolarisations (EAD), a logical step knowing that triggered activity-related arrhythmias are becoming more and more common in hypertrophy and heart failure [5].

## Impulse propagation

For the heart to function properly, excitation and contraction of all myocytes in the heart needs to be coordinated and balanced. Therefore the electrical impulse that initiates excitation moves throughout the heart via a specific route starting in the sinoatrial node. Next the atria are activated, after which the electrical signal passes the atrioventricular node and travels down through the bundle of His and the bundle branches towards the apex of the heart. From there it activates the ventricular myocytes from apex to base via the network of Purkinje fibres and this is further supported by the anisotropic fibre structure. This sequence leads to a coordinated contraction of the ventricles. For the signal to travel from myocyte to myocyte they need to be coupled electrically. This electrical and metabolic coupling of myocytes is facilitated by gap junctions that are built from connexin proteins (Cxs). In the heart three isoforms of Cxs are present. Cx40 is mainly expressed in the atria and throughout the conduction system. Cx43 is the most abundant Cx isoform in both the atria and ventricles but is also found in the distal conduction pathway. Finally, Cx45 is only found in the nodes, the His bundle, and bundle branches [6]. For a detailed review of cardiac connexins see Jansen et al. [6]. The Cxs are localised in the intercalated disks between the myocytes. It is this localisation that facilitates that conduction is anisotropic with a faster conduction along the fibre length (longitudinal conduction) compared with the conduction perpendicular to that given orientation (transverse conduction).

Remodelling of the highly homogeneous expression pattern of Cxs during disease may contribute to generation of a proarrhythmic substrate which may increase the propensity for re-entry based arrhythmias. Re-entry based arrhythmias are, however, beyond the scope of this review. In addition, focal uncoupling of the electrical syncytium may favour the occurrence of ectopic activity. The mechanisms that underlie ectopic activity will be further discussed and provide potential new targets for antiarrhythmic interference.

## Impulse generation and contraction

### Ions → action potential duration (APD)

**Fig. 1:**
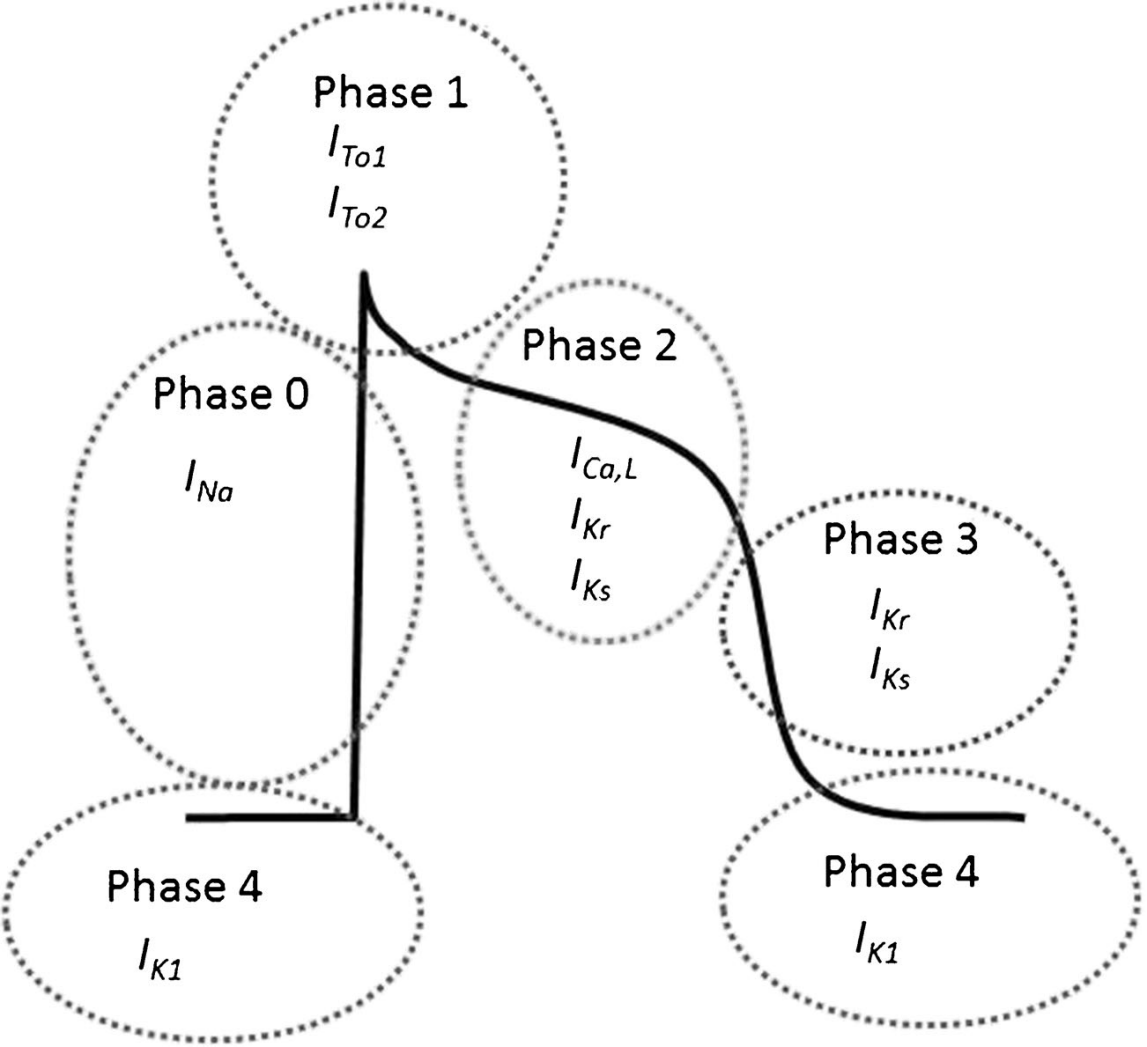
Action potential and ion currents. Phases of the action potential and the responsible ion currents are discussed in the text

The action potential (AP) is generated via a complex interaction of ion channels, and membrane voltage [7], and is generally divided into five phases. They are established through a fine-tuned interaction of sodium (Na^+^), potassium (K^+^), and Ca^2+^ currents. The inward Na^+^ current (*I*_*Na*_) is responsible for the upstroke during phase 0 (Fig. 1, phase 0). The total Na^+^ current is formed by the peak and late *I*_*Na*_: the latter contributes to depolarisation currents during the plateau phase. During phase 1, the Na^+^ channel inactivates considerably and at the same time *I*_*to1*_ and *I*_*to2*_ (transient outward currents) create outward currents of K^+^ and chloride, respectively, to form the notch (Fig. 1, phase 1). Subsequently, Ca^2+^ enters the cell through voltage-gated Ca^2+^channels (L-type calcium channels, LTCC) (*I*_*Ca,L*_) and is involved in creating the plateau phase of the AP since inward movement of Ca^2+^ is counterbalanced by outward K^+^ flow driven by the delayed rectifier potassium currents *I*_*Ks*_ and *I*_*Kr*_ (Fig. 1, phase 2). The plateau phase delays repolarisation of the AP creating time for contraction and relaxation of the cardiomyocytes in between action potentials. Full repolarisation occurs when the LTCC closes and *I*_*Ks*_ and *I*_*Kr*_ take over dominantly (Fig. 1, phase 3). Finally, K^+^ restores the negative membrane potential via the *I*_*K1*_ current (Fig. 1, phase 4) [7].

### Excitation-contraction coupling

**Fig. 2:**
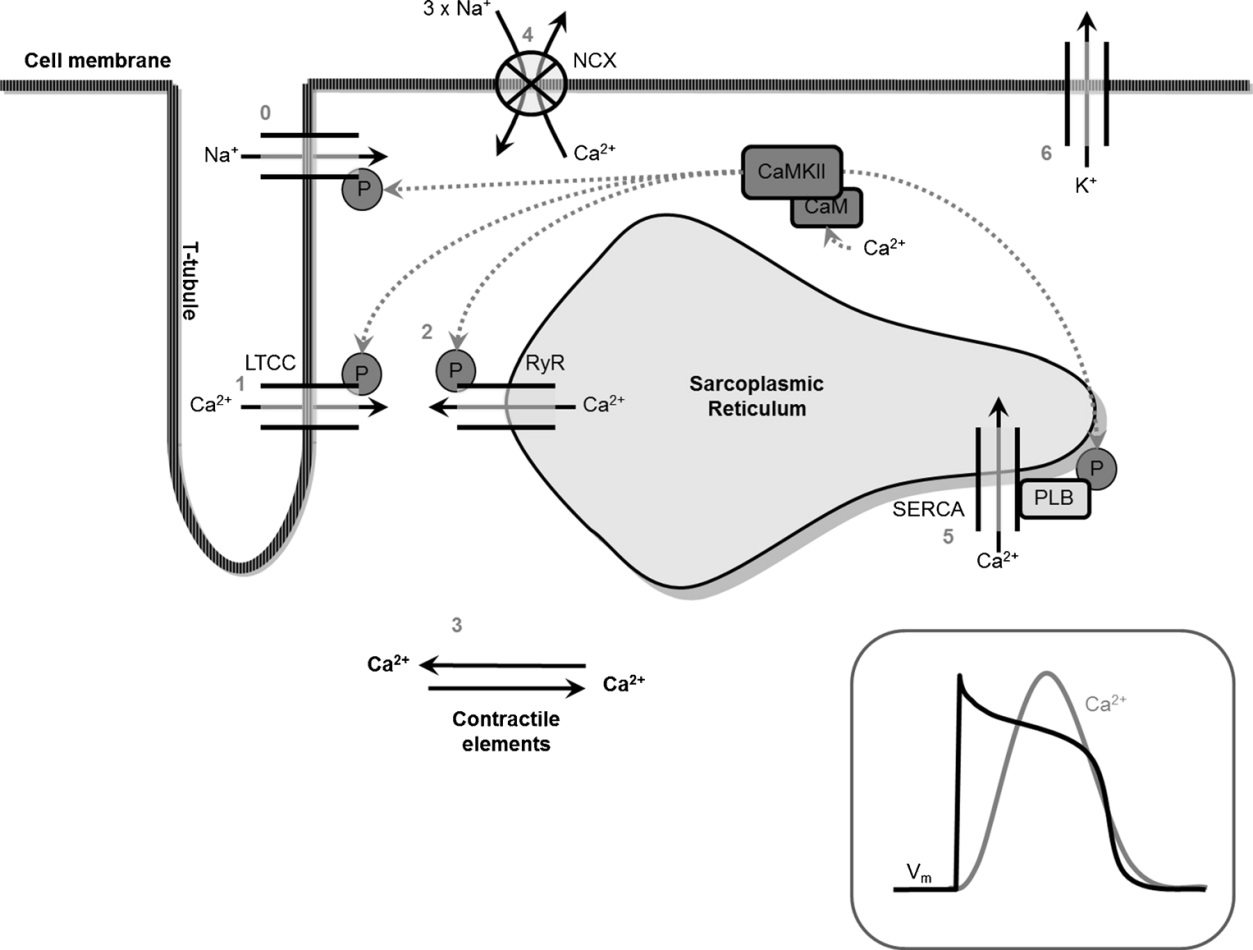
Calcium handling. 0; Sodium enters the cell, creating the AP upstroke. 1; Calcium enters via the LTCC facilitating the plateau phase of the AP and initiating CICR. 2; via RyR on the sarcoplasmic reticulum leading to 3; calcium binding to the contractile elements: excitation-contraction coupling. 4; NCX transports calcium from the cell in exchange for sodium. 5; calcium is pumped back into the SR via SERCA, together with 4 this leads to relaxation of the contractile elements and the end of the plateau phase. 6; Potassium restores the negative membrane potential

Of the ions involved in the activation of the heart, Ca^2+^ plays a key role in excitation-contraction. As mentioned, Ca^2+^ has effects on the membrane potential during the AP plateau via LTCC. LTCC is activated upon depolarisation of the sarcolemma due to a local increase of positive charge that is brought about through influx of [Na^+^], while LTCC is inactivated by local [Ca^2+^]_i_ via calmodulin (CaM) binding on the C-terminus of the channel. The initial Ca^2+^ influx via the LTCC leads to Ca^2+^ induced calcium release (CICR) from the sarcoplasmic reticulum (SR), which is mediated by the RyRs (reviewed by Bers [8]). When RyR is activated, this leads to Ca^2+^ extrusion from the SR thereby increasing [Ca^2+^]_i_ but this increase in Ca^2+^ also triggers inactivation of the LTCC. The RyR is a channel, but also a scaffolding protein that clusters proteins such as CaM (exerts Ca^2+^ dependent modulation of RyR and LTCC function, see below), protein kinase A (PKA, which can alter RyR and *I*_*ca*_ gating), and sorcin (which connects RyRs and LTCCs) near the Ca^2+^ release complex. Subsequently, Ca^2+^ released from the SR binds to troponin to facilitate contraction of the sarcomere, the contractile element of the myocyte. Thus, Ca^2+^ links the electrical activation of cardiomyocytes to mechanical contraction: excitation-contraction coupling (Fig. 2).

During relaxation, free cytoplasmic Ca^2+^ must decline to allow Ca^2+^ to dissociate from troponin leading to relaxation of the contractile element. This Ca^2+^ transport is facilitated by a Ca^2+^-ATPase (SERCA) on the SR which transports Ca^2+^ back into the SR and the NCX on the sarcolemma [8]. SERCA is an active Ca^2+^ pump whose activity is controlled by the phosphorylation status of phospholamban (PLN). When certain residues on PLN are not phosphorylated, SERCA activity is inhibited but this inhibition is relieved when PLN becomes phosphorylated by PKA. Activators of PKA, such as β-adrenergic stimulation, can therefore play a role in relaxation, as more Ca^2+^ is restored in the SR because of higher SERCA activity. This, in turn, renders more Ca^2+^ available for CICR in the subsequent beats which results in a stronger force of contraction.

The NCX on the sarcolemma exchanges three Na^+^ ions for one Ca^2+^ ion. This exchange generates an electrical current that can go in both directions and is dependent on the [Na^+^] and [Ca^2+^] across the sarcolemma as well as the membrane potential. Whether the current is in the forward or reversed mode depends on the driving force for NCX. High [Ca^2+^]_i_ favours forward *I*_*Na/Ca*_ whereas high [Na^+^]_i_ and positive membrane potential favours reversed *I*_*Na/Ca*_ [8].

## Arrhythmias: abnormal excitation

### Triggered arrhythmias

**Fig. 3:**
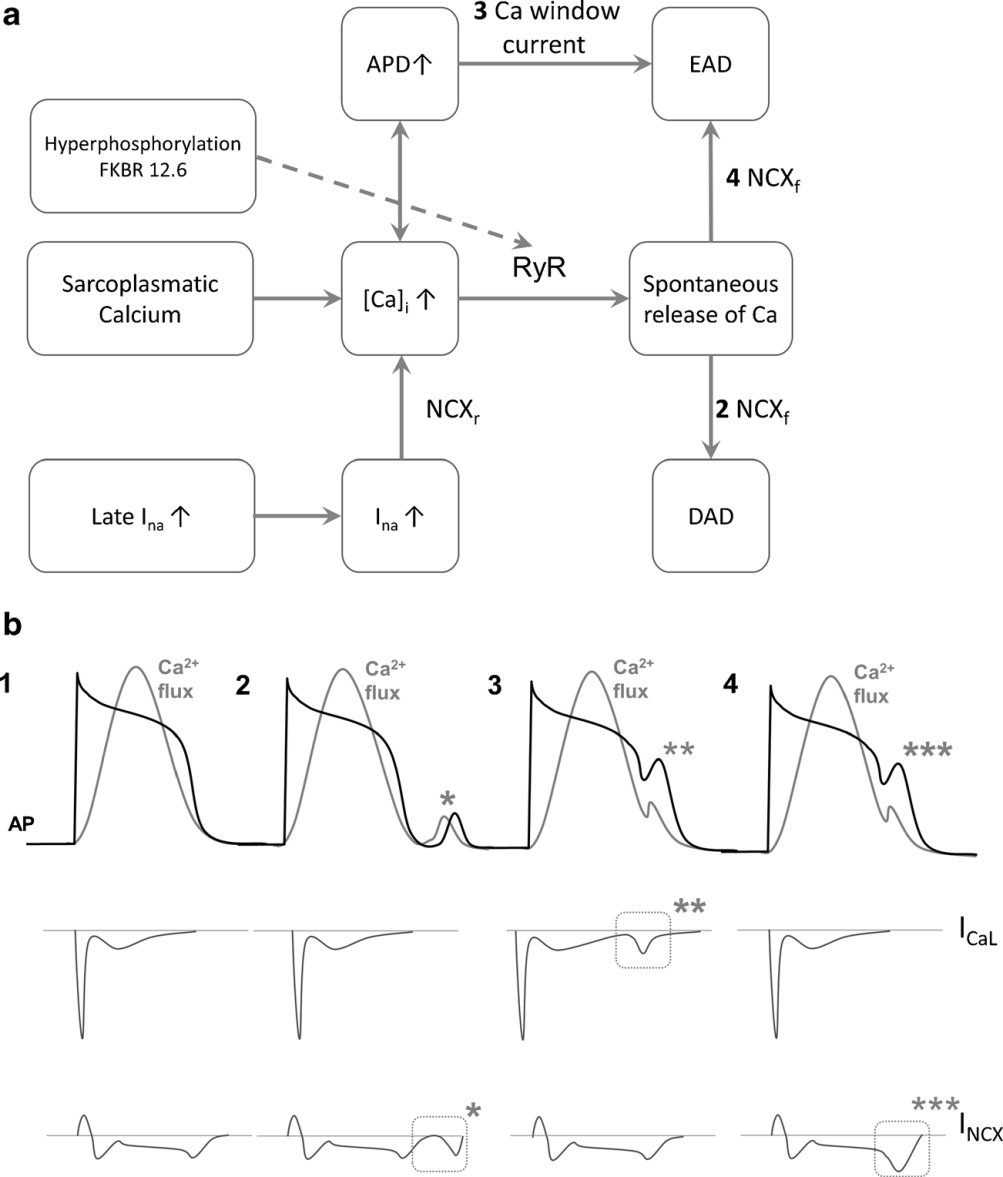
**a** EAD and DAD formation. SR calcium overload leads to increased [Ca^2+^]_i_. This can lead to prolonged action potential duration creating a calcium window current potentially leading to EAD (3). Increased [Ca^2+^]_i_ on the other hand can lead to spontaneous calcium release via CICR resulting in either EADs (4) or DADs (2) via NCX. Also, late *I*_*na*_ is able to increase [Ca]_i_ via NCX_r_, hereby contributing to EAD formation. Numbers corresponding to *black numbers* in **b. b** 1; normal action potential and *I*_*CaL*_ and *I*_*NCX*_. 2; DAD occurring due to forward NCX activity (*). 3; EAD due to calcium window current via LTCC (**). 4; EAD due to forward NCX activity (***)

In hypertrophy and heart failure, Ca^2+^ handling is disturbed. As has been shown in several models, functional expression of SERCA is reduced whereas activity of the NCX is increased [5, 8]. Moreover, kinetics of RyR openings are also changed, leading to unanticipated Ca^2+^releases that can initiate EADs and DADs (Fig. 3). They are defined as: oscillations that attend (EADs) or follow (DADs) the cardiac AP and respond to preceding activation for their manifestation [9]. When the amplitude of the depolarisation reaches threshold, triggered activity in the form of ectopic beats occurs.

### DADs

**Fig. 4:**
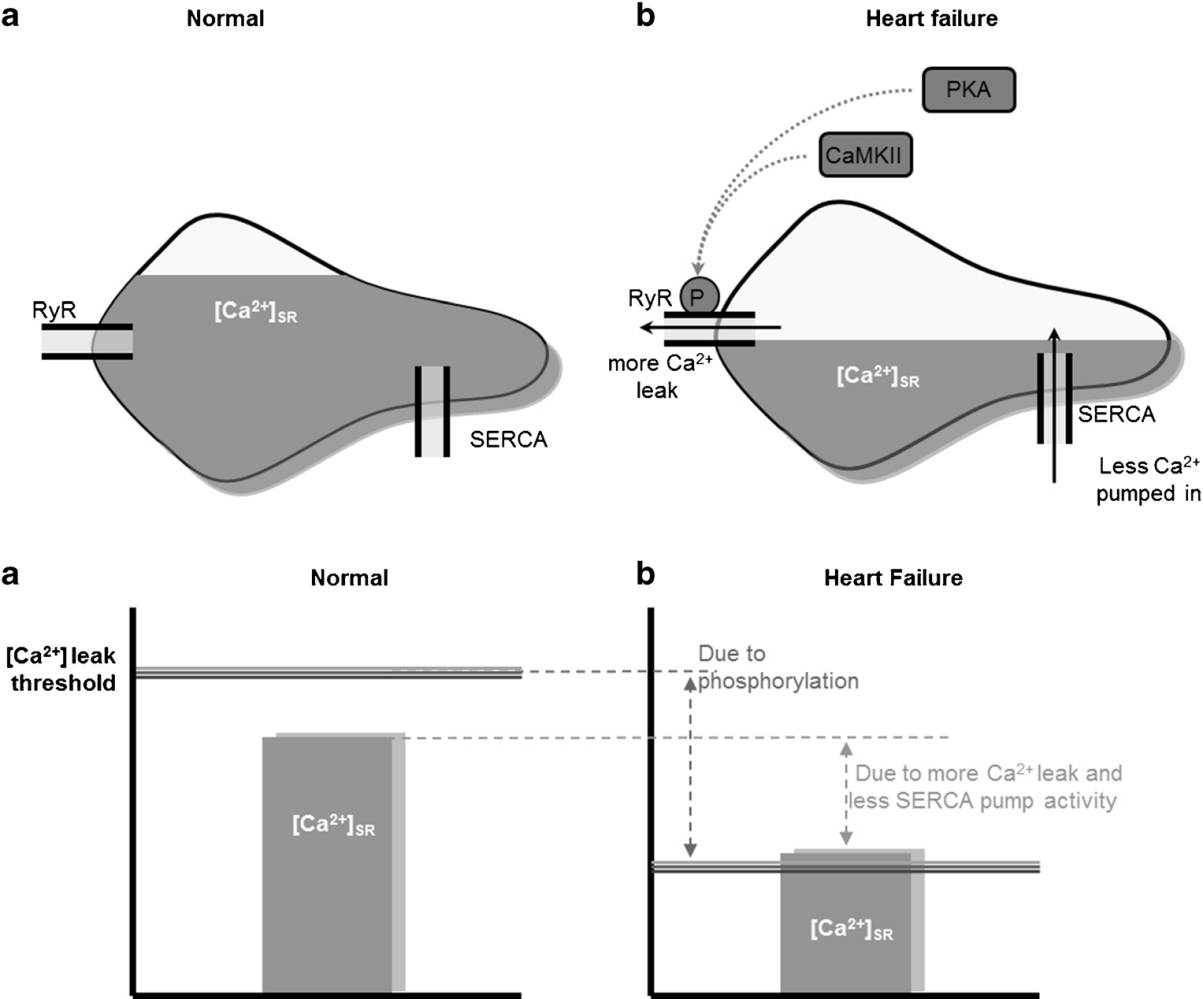
**a** RyR open probability, [Ca^2+^]_SR,_ and sparks. Calcium sparks occur when [Ca^2+^]_SR_ reaches RyR opening threshold. RyR opening threshold is influenced by the open probability of RyR. Higher open probability lowers the threshold. [Ca^2+^]_SR_ is affected by total [Ca^2+^]_in_ and SERCA. **b** In heart failure [Ca^2+^]_SR_ is lowered but the RyR open threshold is lowered more extensively rendering [Ca^2+^]_SR_ higher then threshold

DADs arise after full repolarisation of the myocyte. Ca^2+^ release from the SR is triggered when free SR [Ca^2+^], ([Ca^2+^]_SR_), reaches a certain threshold leading to opening of RyRs (Fig. 4) [10]. [Ca^2+^]_SR_ is influenced by total [Ca^2+^]_i_ and the activity of SERCA. Increased [Ca^2+^]_SR_ load due to higher SERCA activity, for example via β-adrenergic stimulation, brings the [Ca^2+^]_SR_ closer to the threshold for SR leak. Furthermore, the threshold is affected by the RyR open probability: if the open probability increases, the threshold lowers. The open probability of RyR is modulated by [Ca^2+^]_SR_, [Ca^2+^]_i_, AP, RyR phosphorylation and the stabilising protein FKBP12.6 (Calstabin) [11, 12]. RyR phosphorylation is among others executed by CaMKII, and this increases the open probability of RyR [13, 14]. Opening of multiple RyRs creates Ca^2+^ sparks, which can lead to DADs via creation of the transient inward current (*I*_*ti*_) by the NCX (Fig. 3) [15]. If the DADs reach threshold a new AP arises. In heart failure, [Ca^2+^]_SR_ and threshold are both lowered (Fig. 4) yet threshold is affected more than [Ca^2+^]_SR_ leading to a higher occurrence of triggered arrhythmias in these patients [16].

### EADs

EADs are generated in the ventricle during phase 2 or phase 3 of the AP, and can occur during prolongation of AP duration (APD) via a window current of the LTCC (Fig. 3) [9, 17]. However, also NCX plays a role in EAD formation in a mode which is comparable with that as described for DADs. This indicates that EAD formation can occur via two routes, namely a sarcolemmal (LTCC) and an SR (RyR and NCX) dependent mechanism [18]. Whether the LTCC window or NCX has a dominant role in creating EADs depends on the disease setting; it has been suggested that when oxygen radicals are involved LTCC seems dominant, whereas in a β-adrenergic setting NCX may be dominant [18]. Either way NCX and LTCC are both important players in the generation of EADs. In addition, an increase in *I*_*Na-Late*_ leads to higher [Na^+^]_i_, which pushes NCX in its reverse mode and Na^+^ is transported out and Ca^2+^ into the cell, also leading to increased [Ca]_i_ [19]. Via the aforementioned mechanism, this in turn can lead to EAD formation again, as is shown in Fig. 3.

## Old strategies

Antiarrhythmic drugs are still classified according to the Vaughan Williams classification. The underlying molecular strategy includes class I: Na^+^ channel block, class II: β-adrenergic receptor block, class III: K^+^ channel block, class IV: Ca^2+^ channel block, and class V: Na^+^/K^+^ ATPase block [20]. Drugs categorised into these various classes all have the capability of terminating arrhythmias via different strategies. These strategies are reviewed elsewhere [21]. Only class II antiarrhythmic drugs, the β-adrenergic receptor blockers, have proved to reduce mortality [21, 22]. One of the drawbacks of the existing drugs is that most of them temper cardiac performance leading to a negative haemodynamic effect in patients. For example, class I drugs such as flecanide are used in supraventricular arrhythmias but are negative inotropic and also have proarrhythmic effects, which are likely due to excessive conduction slowing and reentry like tachyarrhythmias [20, 21]. Class III drugs prolong the AP, thereby increasing the refractory period, meanwhile terminating arrhythmias based on short APDs such as atrial fibrillation. On the other hand, APD prolongation is a well-established risk factor for long-QT arrhythmias and can lead to ectopic activity through EADs (Fig. 3). Class IV agents, due to their Ca^2+^ channel blocking properties, have negatively inotropic, chronotropic, and dromotropic effects. Class V agents such as digitalis lead to high [Na^+^]_i_ which will activate NCX thereby triggering Ca^2+^overload, and associated ectopic activity (Fig. 3). New antiarrhythmic strategies attempt to find ways to increase antiarrhythmic efficacy while relieving or omitting the adverse side effects.

## New targets for antiarrhythmic treatment

Drugs that interfere with CaMKII, NCX, RyR and its associated stabilising protein FKBP12.6, and the *I*_*Na-Late*_ are potential targets that are currently being investigated in order to achieve clinical applicability. The focus is on interfering with calcium handling of the cardiomyocytes and to become active in the prevention or suppression of VTs. To date, interference on SERCA has not resulted in drug candidates with potential clinical applicability. Since SERCA function in heart failure is considered to be depressed (as observed in several preclinical models of heart failure), it is to note that a non-pharmacological, but viral approach is currently under consideration to restore SERCA function under conditions of heart failure [23].

### Acute modulation of antiarrhythmic targets

#### NCX

NCX and SERCA are the most important players during Ca^2+^ removal from the cytoplasm of the cardiomyocyte. During depolarisation under physiological conditions, NCX contributes marginally to the total inward Ca^2+^ current but most of the time the channel is in its forward mode extruding Ca^2+^ from the cell creating a *I*_*ti*_ (proarrhythmic) [24].

#### NCX inhibition

Known inhibitors of NCX are KB-R7943 and SEA-0400. However, KB-R7943 also inhibits LTCC, *I*_*K*_, and *I*_*K1*_ [25, 26], and thereby KB-R7943 is less specific than SEA-0400 (Table 1) [26]. Blocking NCX theoretically leads to Ca^2+^ accumulation and increased SR Ca^2+^ load, which in turn could lead to adverse effects such as Ca^2+^ sparks [28]. Nonetheless, due to the fact that SEA-0400 probably simultaneously inhibits LTCC, thereby inducing negative inotropic effects, this counteraction possibly preserves cardiac output. Moreover, NCX has a profound role in EAD formation (see section above). Therefore, blocking of NCX potentially exerts antiarrhythmic effects [27]. Recently, our group showed that SEA-0400 effectively prohibited torsades des pointes (TdP) arrhythmia and EAD formation and, as important, without the occurrence of the negative inotropic effects that are typically observed when LTCC is blocked alone [27].

**Table 1:** SEA-0400 effects on ion currents in cardiomyocytes and NCX inhibition by SEA0400 as antiarrhythmic

Target	Action	Model	Dose	Block	Author
I_NCX_	Inhibition	Ventricular myocytes (guinea pig)	1 μM	Forward 82.5 % Reverse: 86.2 %	Tanaka et al. 2002 [25]
I_NCX_	Inhibition	CAVB myocytes	1 μM	Forward 50 % Reverse 66 %	Bourgonje et al. 2013 [27]
I_NCX_	Inhibition	Ventricular myocytes (pig, mouse)	0.3–1 μM	Forward 50 % Reverse 70 %	Ozdemir et al. 2008 [28]
I_NCX_	Inhibition	Ventricular myocytes (dogs)	1 μM	Forward 60 % Reverse 80 %	Birinyi et al. 2005 [29]
I_Ca,L_	Inhibition	Cardiac tissue (dogs)	1 μM	3 %	Nagy et al. 2004 [30]
I_Ca,L_	Inhibition	Ventricular myocytes (pig, mouse)	0.3–1 μM	25 %	Ozdemir et al. 2008 [28]
I_Ca,L_	Inhibition	Ventricular myocytes (guinea pig)	1 μM	9 %	Tanaka et al. 2002 [25]
I_Ca,L_	Inhibition	CAVB myocytes	1 μM	33 %	Bourgonje et al. 2013 [27]
Model	Inhibitor	Inhibitor administration	Dose	Effect	Author
In vivo animal model (CAVB dog)	SEA-0400	After challenge	0.4 and 0.8 mg/kg	0.4 mg/kg decreased TdP episodes 7 ± 4 → 3 ± 4 0.8 abolished TdP incidence	Bourgonje et al. 2013 [27]
Single rabbit ventricular myocytes	SEA-0400	After challenge	2 μM	Abolished EADs	Zhao et al. 2012 [18]
Langendorff perfused rabbit hearts (dofetilide induced arrhythmias)	SEA-0400	Prior to challenge	1 μM	No effect on TdP incidence	Farkas et al. 2009 [31]
Langendorff perfused rabbit hearts (sotalol/veratrinide induced arrhythmias)	SEA-0400	After challenge	1 μM	TdP incidence ↓ (16/18 → 1/18, sotolol, 6/13 →0/13 veratrinide)	Milberg et al. 2008 [32]
Isolated guinea pig myocardium (Ouabain induced arrhythmias)	SEA-0400	Co-administration	1 μM	Arrhythmic contractions ↓ (19/26 → 12/26)	Tanaka et al. 2007 [33]
In vivo animal model (dog ischaemia/reperfusion model and digitalis induced arrhythmias)	SEA-0400	Prior and co-administration (I/R) and After challenge (Digitalis)	0.3–3 mg/Kg	Did not change haemodynamics. No antiarrhythmic effect (I/R). Arrhythmic ratio ↓ (digitalis)	Nagasawa et al. 2005 [34]
Isolated dog Purkinje fibres (dofetilide induced arrhythmias)	SEA-0400	After challenge	1 μM	EAD amplitude ↓ (26.6 ± 2.5 → 14.8 ± 1.8 mV) DAD incidence ↓ (6/6 → 3/6)	Nagy et al. 2004 [30]
In vivo animal model, guinea pigs (aconitine induced arrhythmias)	SEA-0400	Prior to challenge	1–10 mg/Kg	Ineffective in suppressing triggered activity	Amran et al. 2004 [35]

Overview of studies on isolated cardiomyocytes, tissue preparations, whole hearts, and intact animals. *EAD* early after depolarisation. *TdP* torsade de pointes arrhythmia. *I/R* ischaemia reperfusion model. *DAD* delayed after depolarisation

Antiarrhythmic approaches using NCX block do not, however, provide a uniform and straightforward answer about the effectiveness of this strategy (Table 1). In ventricular myocytes SEA-0400 showed different effects on EADs in two different models, although it efficiently inhibited EADs in both settings [18]. In another study, using canine isolated Purkinje fibres, EAD amplitude and DAD incidence were also suppressed by SEA-0400 [30]. Similarly, in Langendorff perfused rabbit hearts, SEA-0400 administration (in the presence of sotalol or veratrinide in order to induce TdP) shortened APD and the incidence of EADs [32], thereby reducing the incidence of arrhythmias [33].

In contrast, in Langendorff perfused rabbit hearts, Farkas et al. observed no antiarrhythmic effects when TdPs where induced using dofetilide [31]. In guinea pigs, no effect of SEA-0400 on arrhythmogeneity was found, and no effect on AP configuration was noticed [35]. On the other hand, in dogs, SEA-0400 did not change heart rate or blood pressure, which are promising findings [27]. Moreover, a 2007 study showed that SEA-0400 had no effects on inotropy and could reverse the positive inotropic effects of Ouabain in isolated guinea pig myocardium [33]. Nevertheless, no antiarrhythmic outcome was found in ischaemia-reperfusion induced arrhythmias.

Concerning safety, SEA-0400 decreased arrhythmias based on intoxication with digitalis, but also caused AV block and cardiac arrest in a small number of the dogs [34]. Due to lack of consensus found in these results more research should be performed before it can be concluded whether or not NCX block has potency in clinical use.

#### RyR

RyR, and its function in cardiac physiology, has been extensively reviewed by Kushnir and Marks [37–39]. Whether or not RyR opens to release Ca^2+^ depends on the open probability of RyR and the SR Ca^2+^ load, which are influenced via various mechanisms, as described earlier.

#### RyR inhibition

Spontaneous release of Ca^2+^ by RyR is involved in the generation of triggered activity (Fig. 3a), and as such RyR is an interesting target in antiarrhythmic therapy that would be based on a decreased risk of DAD formation. This spontaneous release of Ca^2+^ is causative for arrhythmias as found in a disease named catecholaminergic polymorphic ventricular tachycardia (CPVT). Mutations in RyR can enhance the susceptibility for Ca^2+^ leak, especially under conditions with an increased adrenergic drive. Flecainide, a well-established class-Ic antiarrhythmic, showed additional and remarkable efficacy to suppress these arrhythmias both in mice and humans through enhancement of the threshold for triggered activity [40, 41]. It remains to be discussed what the exact contribution is of sodium channel block and RyR inhibition in this preventive effect.

K201 (JTV-519) is a more recent compound that has been tested as a potential inhibitor of RyR. It decreased spontaneous Ca^2+^ release at 1 μM by binding to FKBP12.6, thereby increasing its affinity for RyR and stabilisation of the closed conformation of RyRs [42, 43]. K201 has, however, various other effects on different sarcolemmal ion channels (a multi-channel blocker) [1], see Table 2. Previous experiments have not been able to confirm consistent antiarrhythmic effects of K201 (Table 2). Of the positive studies, K201 prevented the frequency of spontaneous APs after Ouabain in mice with the RyR R4496C+/− mutation [49]. Wehrens et al. showed that when FKBP12.6^+/−^ mice were treated with K201 no arrhythmias could be recorded, which was in contrast to non-treated animals that regularly showed SCD [42]. On the other hand, experiments on isolated cardiomyocytes or in intact RyR2 R4496C knock-in mice, elicited no decrease in DAD incidence upon K201 administration [52]. In the CAVB (chronic AV block) dog, K201 was not able to decrease the incidence nor the severity of dofetilide-induced TdP. In addition, K201 prolonged repolarisation and slowed heart rate, although no negative inotropic effects were observed [48]. These divergent results are in contrast with the well-established molecular role of RyR in the incidence of Ca^2+^ sparks and DADs and its suppression by K201.

**Table 2:** K201 effects on ion currents in cardiomyocytes and experimental models focusing on antiarrhythmic properties of K201s

Target	Action	Model	Dose	Block	Author
I_na_	Inhibition	Ventricular cardiomyocytes (guinea pig)	1.2 μM	50 %	Kimura et al. 1999 [44]
I_K1_	Inhibition	Ventricular cardiomyocytes (guinea pig)	5 μM	50 %	Kimura et al. 1999 [44]
I_Ca_	Inhibition	Ventricular cardiomyocytes (guinea pig)	3 μM	50 %	Kimura et al. 1999 [44]
I_Ca_	Inhibition	Ventricular cardiomyocytes (rabbit)	3 μM	34 %	Loughrey et al. 2007 [43]
I_Kr_	Inhibition	Ventricular cardiomyocytes (guinea pig)	1.2 μM	50 %	Kiriyama et al. 2000 [45]
I_Kr_	Inhibition	Atrial cardiomyocytes (guinea pig)	1 μM	50 %	Nakaya et al. 2000 [46]
I_K-ACH_	Inhibition	Atrial cardiomyocytes (guinea pig)	0.12 μM	50 %	Nakaya et al. 2000 [46]
Model	Inhibitor	Inhibitor administration	Dose	Effect	Author
In vivo rat isoproterenol or I/R induced	K201	Prior to challenge	1 mg/kg	Incidence of arrhythmia ↓ 9/10 →2/10 (isoproterenol) 14/14 → 7/15 (I/R)	Otani et al. 2013 [47]
In vivo dog (CAVB dog)	K201	Prior to and during challenge	0.1 and 0.3 mg/kg/2 min followed by 0.01 and 0.03 mg/kg/30 min iv	No significant anti-arrhythmic but pro-arrhythmic effects were observed	Stams et al. 2011 [48]
RyR^R4496C^ myocytes +/− ouabain	K201	Acutely upon DAD incidence, and prior to challenge	1 μmol/L	No effect on DADs under baseline conditions. But decreased incidence of spontaneous APs during ouabain challenge	Sedej et al. 2010 [49]
Pulmonary cardiomyocytes, isoprenaline (rabbit)	K201	Prior to challenge	0.3 μM	Reduction in spontaneous activity	Chen et al. 2008 [50]
In vivo rabbit methoxamine and clofilium induced	K201	After challenge	50, 200, 400 μg/kg/min	TdP incidence ↓ 6/6 → 4/6, 2/5, 0/6 (dose dependent)	Hasumi et al. 2007 [51]
RyR^R4496C^ myocytes + in vivo mouse (RyR 4496C^+/−^)	K201	Prior to challenge	1 μmol–10 μmol 18 mg/Kg per day	Failed to limit DADs or arrhythmias	Liu et al. 2006 [52]
Mouse whole animal (FKBP12.6^−/−^ en FKBP12.6^+/−^)	K201	Prior to challenge	0.5 mg/kg per hour	Prevention of arrhythmias and SCD in FKBP12.6^+/−^ but not FKBP12.6^−/−^ mice.	Wehrens et al. 2004 [42]
In vivo dog pacing induced	K201	Prior to challenge	0.03 mg/kg/min	AF episodes ↓ 4.2 ± 2.9 → 0 ± 0	Kumagai et al. 2003 [53]
Isolated guinea pig heart	K201	After challenge	0.3 μM and 1 μM	Incidence AF ↓ 2/5 → 0/6	Nakaya et al. 2000 [46]

*DADs* delayed after depolarisations. *AP* action potential. *SR* sarcoplasmic reticulum. *SCD* sudden cardiac death. Adapted from Currie et al. [1]

In the mentioned dog study, our group showed that at a relatively high dose (1.5–2 μM), even proarrhythmic effects of the drug were observed [48]. Although K201 has already been used in clinical trials for treatment of atrial fibrillation (AF), the results seem disappointing: only one has been completed without publication of the results, and two other trials were prematurely terminated.

Other drugs are currently being tested or designed to allow continuation of targeting RyR. Carvedilol, a registered β-blocker, also blocks RyR and prevents spontaneous Ca^2+^ release at doses higher than needed for its β-blocking activity. In this respect, the development of the carvedilol analogue VK-II-86 has to be mentioned since it shows an enhanced specificity regarding the RyR blocking capacities. It has been suggested that in combination with a potent β-blocker, this could be a promising antiarrhythmic approach although thus far no further studies have been published using these compounds in order to test their efficacy [54].

#### CaMKII

Besides its activation under pathological conditions through oxidation by reactive oxygen species (ROS), activation of CaMKII is mainly facilitated by CaM, a small cytoplasmic protein. CaM needs to bind cytosolic Ca^2+^ before CaMKII can be activated [55]. For that, CaMKII activation is indirectly dependent on [Ca^2+^]_i_, but due to its capability of autophosphorylation is not solely dependent on the rise and fall in [Ca^2+^]_i._. When phosphorylation of CaMKII has been accomplished, the enzyme becomes persistently active and therefore the natural beat-to-beat fall in [Ca^2+^]_i_ will not immediately affect the enzymes’ activity. CaMKII has a central role in Ca^2+^ handling, influencing RyR, LTCC, and SERCA (Fig. 2). By phosphorylating RyR, CaMKII increases the open probability [12, 56]. LTCC phosphorylation by CaMKII leads to faster recovery from inactivation [57], whereas the effect of CaMKII on SERCA (via phosphorylation of PLN) leads to an increased SR Ca^2+^ load. CaMKII activation leads to an increase in [Ca^2+^]_i_ and is therefore able to influence NCX by pushing it into the forward mode which results in a depolarising current. All of the above-mentioned actions are under physiological conditions. During cardiac pathology CaMKII expression and function is upregulated, which can trigger proarrhythmia via induction of ectopic activity [58–60]. This makes it an interesting target for antiarrhythmic intervention.

#### CaMKII inhibition

Up till now two CaMKII inhibitors are known, W7 and KN-93. KN-93 is a compound that competes with CaM for the binding site on CaMKII, and through this mode it inhibits activation of CaMKII, with an IC_50_ of 370 nM [61]. However, KN-93 also appears to act as a multi-channel blocker in cardiomyocytes as is depicted in Table 3. W7 is actually an inhibitor of CaM [74], and therefore considered to be an indirect inhibitor of CaMKII as well as of other targets of CaM (e.g. RyR, LTCC).

**Table 3:** KN-93 effects on ion currents in cardiomyocytes and overview of antiarrhythmic experiments with CaMKII inhibitors W7 and KN-93

Target	Action	Model	Dose	Block	Author
*I*_*ca*_	Inhibition	Rabbit cardiomyocytes	1 μM	41 %	Anderson et al. 1998 [62]
*I*_*K*_	Inhibition	HEK cells overexpressing Kv1,5	3 μM	50 %	Rezazadeh et al. 2006 [63]
Model	Inhibitor	Inhibitor administration	Dose	Effect	Author
In vivo animal model (mdx mice)	KN-93	After challenge	1 μM	VT incidence ↓ in presence of KN-93 (7/14 → 2/14)	Ather 2013 [64]
CAVB dog (dofetilide induced)	W7	After challenge	18.87 mg/kg/5 min	Abolished almost all TdPs	Bourgonje et al. 2012 [36]
Langendorff perfused rat heart (AngII induced arrhythmia)	KN-93	Prior to challenge	2 μM	VF incidence ↓ in presence of KN-93 (4/4 → 1/4)	Bapat et al. 2012 [65]
Langendorff perfused rat heart	KN-93	Prior to challenge	2.5 μM	Incidence of premature beats ↓ (71.5 %)	Said et al. 2011 [66]
Langendorff perfused rat heart (glycolytic inhibition induced arrhythmia)	KN-93	Prior to and after challenge	1 μM	Incidence of VT/VF ↓ (6/6 → 4/9)	Morita et al. 2011 [67]
Whole animal (mouse RyR_2_-S2814D knock in and aortic banding)	KN-93	Prior to challenge	30 μmol/kg	Incidence of VT ↓ (6/12 → 0/12)	Lui et al. 2011 [68]
Whole animal (heart failure mouse, iso induced arrhythmia)	KN-93	Prior to challenge	20 μmol/L/kg	Incidence of arrhythmias ↓ (5/6 → 0/4)	Sag et al. 2009 [69]
Langendorff perfused rabbit heart	W7	Co-administration with challenge	20, 50, 100 μM	EAD incidence ↓ (9/9 → 1/9) TdP incidence ↓ (7/9 → 1/9)	Pu et al. 2005 [70]
Langendorff perfused mouse heart	W7, KN-93	After challenge	W7: 25 μM KN–93: 2 μM	W7: pVT incidence ↓ (7/11 → 1/11) KN93: pVT incidence ↓ (5/8 → 1/8)	Kirchhof et al. 2004 [71]
In vivo animal model (methoxamine rabbit model)	W7	Prior to challenge	50 μM/kg	TdP incidence ↓ (12/14 → 1/11)	Gbadebo et al. 2002 [72]
In vivo animal model (methoxamine rabbit model)	W7	Prior to challenge	25, 50 μM/kg	TdP incidence ↓ (6/8 → 1/7)	Mazur et al. 1999 [73]
Langendorff perfused rabbit heart	KN-93	Prior to challenge	0.5 μM	EAD Incidence ↓ (8/8 → 4/10)	Anderson et al. 1998 [62]

*AV* atrioventricular node; *TdP* torsades des pointes; *AngII* angiotensin II; *VT* ventricular tachycardia; *VF* ventricular fibrillation; iso: isoproterenol; *EAD* early after depolarisation; *pVT* polymorphic VT

So far, CaMKII antagonists show promising experimental results (Table 3). In genetically engineered Langendorff perfused mouse hearts, W7 and KN-93 suppressed VTs in almost all mice. However, W7 appeared to prolong APD to a higher degree than KN-93 which renders the use of the more specific inhibitor KN-93 favourable in this setting [71]. In Langendorff perfused rat hearts, KN-93 effectively suppressed ventricular fibrillation (VF) without affecting APD [67], and also lowered the incidence of premature beats occurring after ischaemia reperfusion interventions [66]. Similarly, VF induced by angiotensin II was prevented by KN-93 in 75 % of the hearts [65]. Furthermore, KN-93 also prevented the occurrence of EADs in Langendorff perfused rabbit hearts [62], while W-7 decreased EAD as well as TdP incidence, even though W-7 was not able to counteract the sotalol-induced APD prolongation [70].

Looking at intact animal studies, CaMKII inhibition by KN-93 was able to prevent isoproterenol-induced arrhythmias [69], as well as in other experimental setups in mice, such as aortic banding, where KN-93 also proved to be successful in preventing VTs [68]. In rabbits, pretreatment with W-7 prevented methoxamine-induced TdP [72, 73], and had no undesirable effects on haemodynamics [73]. In CAVB dogs, W-7 was successful in terminating dofetilide-induced TdP in the majority of dogs [36]. Given its antiarrhythmic potency, the minimal effects on APD and the fact that haemodynamics were virtually unaffected, CaMKII inhibition might be a favourable approach when compared with the old antiarrhythmic strategies.

#### Late inward sodium current

The total inward sodium current is composed of a peak and late component and is provided through voltage-gated Na channels in the sarcolemma, which are rapidly activated and inactivated. Under physiological conditions, the *I*_*Na-Late*_ has a much smaller amplitude than peak *I*_*Na*_. Under pathological circumstances, however, the *I*_*Na -Late*_ can be elevated up to 5 times, and substantially affect total *I*_*Na*_ leading to accumulation of Na^+^ in the cytosol [75]. On its own, increased [Na]_i_ leads to APD prolongation, and by pushing NCX in the reverse mode it can lead to increased [Ca^2+^]_i_, and triggered arrhythmias.

#### Late I_Na_ inhibition

Due to its previously described role in arrhythmias, inhibition of *I*_*Na-Late*_ is a topic of interest because it can possibly prevent EAD and DAD triggered activity. Ranolazine is a well-known inhibitor of *I*_*Na-Late*_ but its actions are not restricted to this channel as it evokes multiple other effects (Table 4). In Table 5, we have listed its antiarrhythmic properties. In Langendorff perfused guinea pig hearts, ranolazine abolished and prevented ATX-II induced (pro) arrhythmic features on APD, EADs and VTs in the ‘LQT-3 syndrome’ [84]. In guinea pig isolated ventricular myocytes, ranolazine was able to counteract the arrhythmic effects, such as APD prolongation and subsequent EAD formation [82, 86]. In canine wedge preparations, ranolazine also effectively prevented TdP and EADs [76], and in rats it showed various antiarrhythmic properties [77, 80, 85]. Even in clinical trials, ranolazine showed its capability of reducing arrhythmic events [81]. Ranolazine is already being prescribed to treat patients with chronic angina and, importantly, in a clinical trial a significant decrease in the occurrence of ventricular arrhythmias was observed compared to controls [81].

**Table 4:** Ranolazine effects on ion currents in cardiomyocytes

Target	Action	Model	Dose	Block	Author
Late *I*_*Na*_	Inhibition	Canine wedge preparations	6 μmol/L	50 %	Antzelevitch 2004 [76]
Late *I*_*Ca*_	Inhibition	Canine wedge preparations	2–6 μmol/L	25–30 %	Antzelevitch 2004 [76]
*I*_*ks*_	Inhibition	Canine wedge preparations	30 μmol/L	17 %	Antzelevitch 2004 [76]
*I*_*Na-Ca*_	Inhibition	Canine wedge preparations	50 μmol/L	50 %	Antzelevitch 2004 [76]
*I*_*kr*_	Inhibition	Canine wedge preparations	12 μmol/L	50 %	Antzelevitch 2004 [76]

**Table 5:** Late I_na_ inhibition and antiarrhythmic properties

Model	Inhibitor	Inhibitor administration	Dose	Effect	Author
Langendorff perfused rat hearts (rapid pacing induced VF and oxidative stress induced VF)	Ranolazine	Prior to challenge	10 μM	Pacing induced VF shortening >3 min → 12 ± 6 s Oxidative stress induced VF termination and suppression	Morita 2011 [77]
Transgenic CaMKII mice papillary muscles	Ranolazine	After challenge	5 μmol/L	Termination of premature arrhythmogenic contractions	Sossalla 2011 [78]
CAVB dog, dofetilide induced	Ranolazine	After challenge	4 mg/kg/0.5 min + 0.225 mg/kg/min	TdP episodes ↓ 10 → 3	Antoons 2010 [79]
In vivo animal model (rats I/R induced arrhythmias and ischemia induced arrhythmias)	Ranolazine	After challenge (I/R) Prior to challenge (I)	10 mg/kg iv bolus (I/R) 2, 6, 10 μM (I and I/R)	Sustained VT incidence ↓ 9/12 vs. 1/11 (I/R) VF incidence ↓ 10/12, 8/12, 5/10, 4/12 (control, 2, 8, 10 μM Ranolazine resp.)	Dhalla 2009 [80]
Clinical trial	Ranolazine	Prior to challenge		Reduced the incidence of VT vs placebo	Scirica 2007 [81]
Rabbit and guinea pig isolated ventricular myocytes H_2_O_2_ challenge	Ranolazine	After challenge	10 μM	Suppression of APD prolongation and EAD formation	Song 2006 [82]
Canine myocytes of normal and HF dogs	Ranolazine	After challenge	5, 10, 20 μM	Shortening of APD and suppression of EADs	Undrovinas 2006 [83]
Langendorff perfused guinea pig hearts. ATX-II induced arrhythmias	Ranolazine	Both	5 μM	Ranolazine abolished ATX-II induced EADs/VTs and prevented ATX-II induced EADs/VTs in pretreated hearts	Wu 2004 [84]
Langendorff perfused rat hearts I/R ATX-II challenge	Ranolazine	Prior to challenge	4 μM, 9 μM in perfusate	Reduced Ca^2+^ overload and LV mechanical dysfunction	Fraser 2006 [85]
Isolated canine wedge preparations, M cells and Purkinje fibres	Ranolazine	Prior to challenge	1–100 μmol/L	Abolished TdP and EADs	Antzelevitch 2004 [76]
Isolated guinea pig ventricular myocytes–ATX-II challenge	Ranolazine	After challenge	0.1–30 μmol/L	Reduced ATX-II induced EADs	Song 2004 [86]
Canine Purkinje fibres E-4031, ATX-II and high Ca+ isoproterenol induction	GS-967	After challenge	30 nM/100 nM	EAD and DAD incidence ↓ EAD 4/4 → 2/5 → 0/5 (E-4031) EAD 4/4 → 1/4 → 0/4 (ATX-II) DAD 4/4 → 2/4 → 0/5 (high Ca+ isoproterenol)	Sicouri et al. 2013 [87]
Langendorff perfused rabbit heart ATX-II and E-4031 induction	GS-967	After challenge	100 and 600 nmol/L (ATX-II and E-4031 resp.)	Incidence of VT ↓ 6/11 → 0/11 (ATX-II) 5/5 → 0/5 (E-4031)	Belardinelli et al. 2013 [88]
In vivo animal model (rabbits clofilium/methoxamine and ischaemia induced)	GS-967	Prior to challenge	60 μg/kg bolus + 16 μg/kg/min (clofilium) 15 μg/kg + 4 μg/kg/min (ischaemia)	Incidence VT ↓ 5/6 → 1/6 (clofilium) 5/10 → 2/8 (ischemia)	Belardinelli et al. 2013 [88]
Langendorff perfused guinea pig heart isoprenaline induction	Sophocarpine	After challenge	300 μmol/L	incidence VT ↓ 6/6 → 0/6	Yang et al. 2011 [89]

*EAD* early after depolarisation. *TdP* torsade de pointes arrhythmia. *I/R* ischaemia reperfusion model. *VT* ventricular tachycardia. A H_2_O_2_ challenge mimics oxidative stress

Recently, data have been published which suggest that sophocarpine also possesses *I*_*Na-Late*_ inhibiting potency next to its effect on other currents (*I*_*Kr,*_ *I*_*Ca*_) [90]. GS-967 is another new compound that is proposed to inhibit late *I*_*Na,late*_ specifically (it affects peak *I*_*Na*_ and *I*_*Kr*_ only weakly at much higher doses). This new compound has been tested in animal models with positive findings [87, 88], which makes it an interesting and potentially antiarrhythmic compound for future testing in a clinical setting.

## Conclusion

**Fig. 5:**
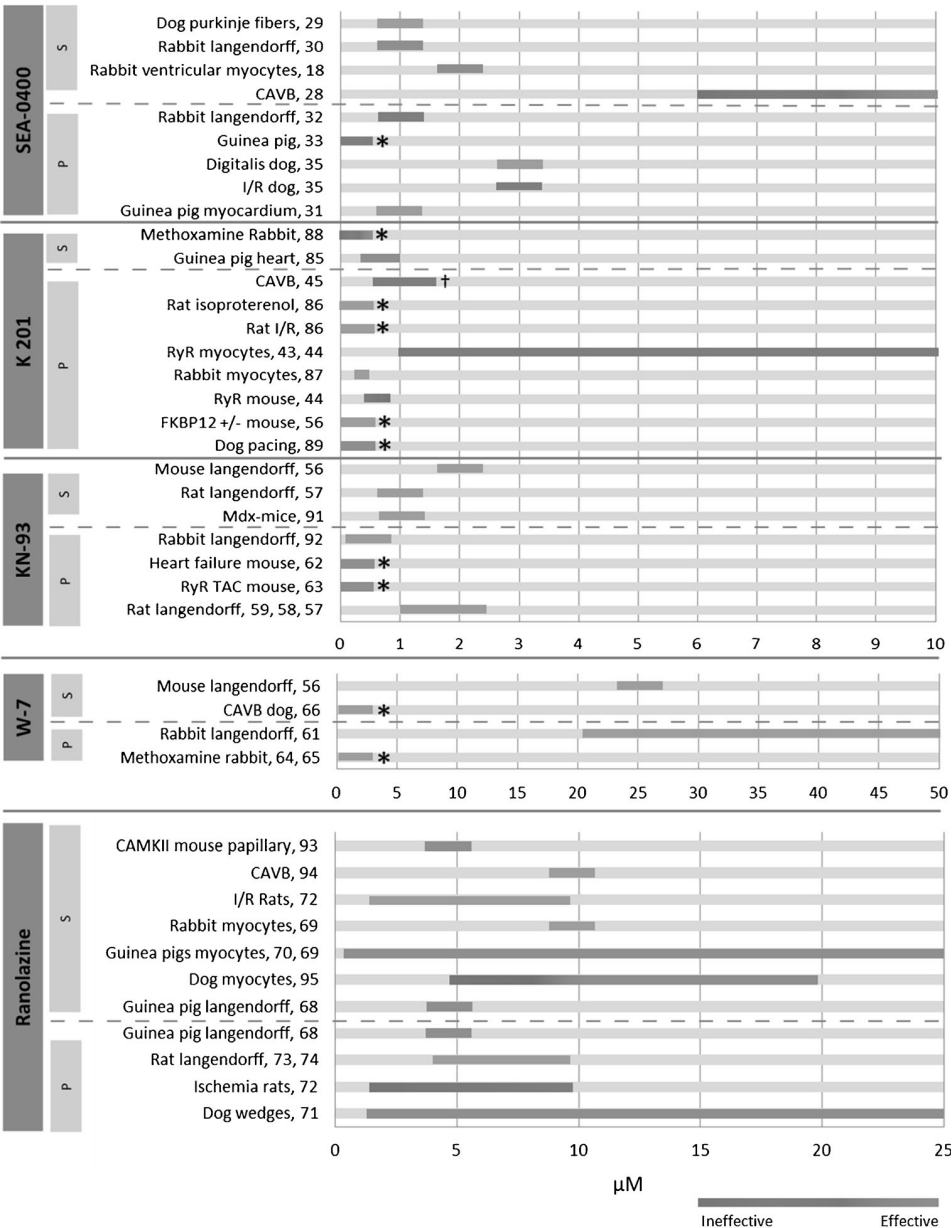
Schematic overview of experimental approaches that have been performed to test efficacy and antiarrhythmic potency of drugs targeting NCX, RyR, CamKII and late I_*Na*_. References are stated behind the model. *S* stands for suppressive, *P* stands for preventive. *Asterisk* indicates papers in which no plasma concentration was measured. *Dagger* indicates model in which proarrhythmic events were observed

Old antiarrhythmic strategies, though proven clinically effective, have important drawbacks such as negative haemodynamic effects and a small safety margin, thereby prohibiting the use of higher dosages [21]. To improve specificity and efficacy of pharmacological intervention, there is a continuous quest for new pharmacological targets. Calcium handling within the cardiomyocytes is such an important target since disturbed calcium handling has a maladaptive and dual effect that leads to both an increased propensity to develop arrhythmias and to induction of contractile dysfunction. In the current study we have reviewed the recent investments made to target NCX, RyR, CaMKII and the late sodium current, all being involved in proarrhythmia due to disturbed calcium handling. Figure 5 provides a schematic overview of the current knowledge regarding the potency and potential efficacy of drugs affecting these new antiarrhythmic targets.

### NCX

So far, blocking of NCX, using SEA-0400, appears to be neutral on inotropy due to the counteracting effects of 1) anticipated positive inotropy by NCX block alone and 2) negative haemodynamics by its additional inhibition of LTCC [27, 36]. Taking into account that this compound is possibly totally specific for NCX, it shows a neutral haemodynamic effect but so far did not prove to be antiarrhythmic in all experimental studies. The pharmacological industry has no plans for further exploration in this direction and therefore the future of this drug as an antiarrhythmic agent is uncertain. Development of new NCX blockers is, however, foreseen and could become tools for future clinical applicability.

### RyR

The antiarrhythmic potential of RyR block remains debatable, as positive results of K201 are scarce and counteracted by negative results (Table 2). Together with the possible proarrhythmic effects at the high dosage that is required for treatment of atrium fibrillation, the lack of convincing antiarrhythmic results, and various effects on other ion currents, K201 may not be the best suitable future antiarrhythmic agent. For this reason, development of a more selective inhibitor is required to assess its antiarrhythmic properties.

### CaMKII

In Table 3, studies on CaMKII inhibition using either W-7 or KN-93 in intact animals and Langendorff perfused hearts are summarised, mostly showing a profound antiarrhythmic effect of the drugs. Also in various experimental settings using isolated cardiomyocytes, the antiarrhythmic properties of CaMKII inhibition are readily observed [36, 91]. Additionally important, blocking CaMKII provides antihypertrophic effects and maintenance of LV function [1]. The clinical use of W-7 and KN-93 is still under consideration. The currently used and obviously attractive drugs are not completely target specific, and long-term inhibition of CaMKII with these drugs induces neurotoxicity [92] which makes them less favourable [93]. Current development of new entities by several industries is expectantly awaited by the scientific community.

### Late *I*_*Na*_ inhibition

The use of ranolazine has also shown positive results: prevention and suppression of EADs, and VT/VF in several experimental approaches. Important aspect is that the drug is already registered and clinically applicable. Its antiarrhythmic efficacy is not complete, which opens the road for improvement using this approach. In this line of intervention, promising new drugs such as sophocarpine and GS-967 are being investigated.

Overall, regardless of the current possibilities for pharmacological interventions, it is clear that new strategies will be welcome to prevent SCD due to arrhythmias. The new targets discussed here prove to be interesting strategies for the future and provide food for thought. Obviously, more research with respect to the long-term effects of chronically blocking each of the discussed targets is essential before research can proceed to a more clinical setting.

## References

[1] Currie S, Elliott EB, Smith GL (2011). Two candidates at the heart of dysfunction: the ryanodine receptor and calcium/calmodulin protein kinase ii as potential targets for therapeutic intervention-an in vivo perspective. Pharmacol Ther.

[2] Huikuri HV, Castellanos A, Myerburg RJ (2001). Sudden death due to cardiac arrhythmias. N Engl J Med.

[3] Sen-Chowdhry S, McKenna WJ (2012). Sudden death from genetic and acquired cardiomyopathies. Circulation.

[4] Kamath GS, Mittal S (2008). The role of antiarrhythmic drug therapy for the prevention of sudden cardiac death. Prog Cardiovasc Dis.

[5] Antoons G, Oros A, Bito V (2007). Cellular basis for triggered ventricular arrhythmias that occur in the setting of compensated hypertrophy and heart failure: considerations for diagnosis and treatment. J Electrocardiol.

[6] Jansen JA, van Veen TA, de Bakker JM (2010). Cardiac connexins and impulse propagation. J Mol Cell Cardiol.

[7] Rudy Y (2008). Molecular basis of cardiac action potential repolarization. Ann N Y Acad Sci.

[8] Bers DM (2002). Cardiac excitation-contraction coupling. Nature.

[9] Burashnikov A, Antzelevitch C (2006). Late-phase 3 ead. A unique mechanism contributing to initiation of atrial fibrillation. Pacing Clin Electrophysiol.

[10] Diaz ME, Trafford AW, O’Neill SC (1997). Measurement of sarcoplasmic reticulum ca2+ content and sarcolemmal ca2+ fluxes in isolated rat ventricular myocytes during spontaneous ca2+ release. J Physiol.

[11] Fink M, Noble PJ, Noble D (2011). Ca(2)(+)-induced delayed after depolarizations are triggered by dyadic subspace ca2(2)(+) affirming that increasing serca reduces after contractions. Am J Physiol Heart Circ Physiol.

[12] Guo T, Zhang T, Mestril R (2006). Ca2+/calmodulin-dependent protein kinase ii phosphorylation of ryanodine receptor does affect calcium sparks in mouse ventricular myocytes. Circ Res.

[13] Lederer WJ, Tsien RW (1976). Transient inward current underlying arrhythmogenic effects of cardiotonic steroids in purkinje fibres. J Physiol.

[14] Mechmann S, Pott L (1986). Identification of na-ca exchange current in single cardiac myocytes. Nature.

[15] Antoons G, Willems R, Sipido KR (2012). Alternative strategies in arrhythmia therapy: evaluation of na/ca exchange as an anti-arrhythmic target. Pharmacol Ther.

[16] Pogwizd SM, Bers DM (2004). Cellular basis of triggered arrhythmias in heart failure. Trends Cardiovasc Med.

[17] Volders PG, Vos MA, Szabo B (2000). Progress in the understanding of cardiac early after depolarizations and torsades de pointes: time to revise current concepts. Cardiovasc Res.

[18] Zhao Z, Wen H, Fefelova N (2012). Revisiting the ionic mechanisms of early after depolarizations in cardiomyocytes: predominant by ca waves or ca currents?. Am J Physiol Heart Circ Physiol.

[19] Noble D, Noble PJ (2006). Late sodium current in the pathophysiology of cardiovascular disease: consequences of sodium-calcium overload. Heart.

[20] Zipes DP, Camm AJ, Borggrefe M (2006). Acc/aha/esc 2006 guidelines for management of patients with ventricular arrhythmias and the prevention of sudden cardiac death: a report of the american college of cardiology/american heart association task force and the european society of cardiology committee for practice guidelines (writing committee to develop guidelines for management of patients with ventricular arrhythmias and the prevention of sudden cardiac death): developed in collaboration with the european heart rhythm association and the heart rhythm society. Circulation.

[21] Gjesdal K (2009). Non-investigational antiarrhythmic drugs: long-term use and limitations. Expert Opin Drug Saf.

[22] Kendall MJ (2000). Clinical trial data on the cardioprotective effects of beta-blockade. Basic Res Cardiol.

[23] Jessup M, Greenberg B, Mancini D (2011). Calcium upregulation by percutaneous administration of gene therapy in cardiac disease (cupid): a phase 2 trial of intracoronary gene therapy of sarcoplasmic reticulum ca2+-atpase in patients with advanced heart failure. Circulation.

[24] Sipido KR, Bito V, Antoons G (2007). Na/ca exchange and cardiac ventricular arrhythmias. Ann N Y Acad Sci.

[25] Tanaka H, Nishimaru K, Aikawa T (2002). Effect of sea0400, a novel inhibitor of sodium-calcium exchanger, on myocardial ionic currents. Br J Pharmacol.

[26] Matsuda T, Arakawa N, Takuma K (2001). Sea0400, a novel and selective inhibitor of the na+-ca2+ exchanger, attenuates reperfusion injury in the in vitro and in vivo cerebral ischemic models. J Pharmacol Exp Ther.

[27] Bourgonje VJ, Vos MA, Ozdemir S (2013). Combined na(+)/ca(2+) exchanger and l-type calcium channel block as a potential strategy to suppress arrhythmias and maintain ventricular function. Circ Arrhythm Electrophysiol.

[28] Ozdemir S, Bito V, Holemans P (2008). Pharmacological inhibition of na/ca exchange results in increased cellular ca2+ load attributable to the predominance of forward mode block. Circ Res.

[29] Birinyi P, Acsai K, Banyasz T (2005). Effects of sea0400 and kb-r7943 on na+/ca2+ exchange current and l-type ca2+ current in canine ventricular cardiomyocytes. Naunyn Schmiedeberg’s Arch Pharmacol.

[30] Nagy ZA, Virag L, Toth A (2004). Selective inhibition of sodium-calcium exchanger by sea-0400 decreases early and delayed after depolarization in canine heart. Br J Pharmacol.

[31] Farkas AS, Makra P, Csik N (2009). The role of the na+/ca2+ exchanger, i(na) and i(cal) in the genesis of dofetilide-induced torsades de pointes in isolated, av-blocked rabbit hearts. Br J Pharmacol.

[32] Milberg P, Pott C, Fink M (2008). Inhibition of the na+/ca2+ exchanger suppresses torsades de pointes in an intact heart model of long qt syndrome-2 and long qt syndrome-3. Heart Rhythm.

[33] Tanaka H, Shimada H, Namekata I (2007). Involvement of the na+/ca2+ exchanger in ouabain-induced inotropy and arrhythmogenesis in guinea-pig myocardium as revealed by sea0400. J Pharmacol Sci.

[34] Nagasawa Y, Zhu BM, Chen J (2005). Effects of sea0400, a na+/ca2+ exchange inhibitor, on ventricular arrhythmias in the in vivo dogs. Eur J Pharmacol.

[35] Amran MS, Hashimoto K, Homma N (2004). Effects of sodium-calcium exchange inhibitors, kb-r7943 and sea0400, on aconitine-induced arrhythmias in guinea pigs in vivo, in vitro, and in computer simulation studies. J Pharmacol Exp Ther.

[36] Bourgonje VJ, Schoenmakers M, Beekman JD (2012). Relevance of calmodulin/camkii activation for arrhythmogenesis in the av block dog. Heart Rhythm.

[37] Kushnir A, Marks AR (2010). The ryanodine receptor in cardiac physiology and disease. Adv Pharmacol.

[38] Dulhunty AF, Casarotto MG, Beard NA (2011). The ryanodine receptor: a pivotal ca2+ regulatory protein and potential therapeutic drug target. Curr Drug Targets.

[39] Ather S, Respress JL, Li N (1832). Alterations in ryanodine receptors and related proteins in heart failure. Biochim Biophys Acta.

[40] Watanabe H, Chopra N, Laver D (2009). Flecainide prevents catecholaminergic polymorphic ventricular tachycardia in mice and humans. Nat Med.

[41] Watanabe H, Steele DS, Knollmann BC (2011). Mechanism of antiarrhythmic effects of flecainide in catecholaminergic polymorphic ventricular tachycardia. Circ Res.

[42] Wehrens XH, Lehnart SE, Reiken SR (2004). Protection from cardiac arrhythmia through ryanodine receptor-stabilizing protein calstabin2. Science.

[43] Loughrey CM, Otani N, Seidler T (2007). K201 modulates excitation-contraction coupling and spontaneous ca2+ release in normal adult rabbit ventricular cardiomyocytes. Cardiovasc Res.

[44] Kimura J, Kawahara M, Sakai E (1999). Effects of a novel cardioprotective drug, jtv-519, on membrane currents of guinea pig ventricular myocytes. Jpn J Pharmacol.

[45] Kiriyama K, Kiyosue T, Wang JC (2000). Effects of jtv-519, a novel anti-ischaemic drug, on the delayed rectifier k+ current in guinea-pig ventricular myocytes. Naunyn Schmiedeberg’s Arch Pharmacol.

[46] Nakaya H, Furusawa Y, Ogura T (2000). Inhibitory effects of jtv-519, a novel cardioprotective drug, on potassium currents and experimental atrial fibrillation in guinea-pig hearts. Br J Pharmacol.

[47] Otani N, Matsuda R, Oda K (2013). Protective effect of k201 on isoproterenol-induced and ischemic-reperfusion-induced ventricular arrhythmias in the rat: comparison with diltiazem. J Cardiovasc Pharmacol Ther.

[48] Stams TR, Oros A, der Nagel R (2011). Effects of k201 on repolarization and arrhythmogenesis in anesthetized chronic atrioventricular block dogs susceptible to dofetilide-induced torsade de pointes. Eur J Pharmacol.

[49] Sedej S, Heinzel FR, Walther S (2010). Na+-dependent sr ca2+ overload induces arrhythmogenic events in mouse cardiomyocytes with a human cpvt mutation. Cardiovasc Res.

[50] Chen YJ, Chen YC, Wongcharoen W (2008). Effect of k201, a novel antiarrhythmic drug on calcium handling and arrhythmogenic activity of pulmonary vein cardiomyocytes. Br J Pharmacol.

[51] Hasumi H, Matsuda R, Shimamoto K (2007). K201, a multi-channel blocker, inhibits clofilium-induced torsades de pointes and attenuates an increase in repolarization. Eur J Pharmacol.

[52] Liu N, Colombi B, Memmi M (2006). Arrhythmogenesis in catecholaminergic polymorphic ventricular tachycardia: insights from a ryr2 r4496c knock-in mouse model. Circ Res.

[53] Kumagai K, Nakashima H, Gondo N (2003). Antiarrhythmic effects of jtv-519, a novel cardioprotective drug, on atrial fibrillation/flutter in a canine sterile pericarditis model. J Cardiovasc Electrophysiol.

[54] Zhou Q, Xiao J, Jiang D (2011). Carvedilol and its new analogs suppress arrhythmogenic store overload-induced ca2+ release. Nat Med.

[55] Lou LL, Lloyd SJ, Schulman H (1986). Activation of the multifunctional ca2+/calmodulin-dependent protein kinase by autophosphorylation: Atp modulates production of an autonomous enzyme. Proc Natl Acad Sci U S A.

[56] Anderson ME (2005). Calmodulin kinase signaling in heart: an intriguing candidate target for therapy of myocardial dysfunction and arrhythmias. Pharmacol Ther.

[57] Guo J, Duff HJ (2006). Calmodulin kinase ii accelerates l-type ca2+ current recovery from inactivation and compensates for the direct inhibitory effect of [ca2+]i in rat ventricular myocytes. J Physiol.

[58] Zhang T, Johnson EN, Gu Y (2002). The cardiac-specific nuclear delta(b) isoform of ca2+/calmodulin-dependent protein kinase ii induces hypertrophy and dilated cardiomyopathy associated with increased protein phosphatase 2a activity. J Biol Chem.

[59] Anderson ME (2009). Camkii and a failing strategy for growth in heart. J Clin Invest.

[60] Zhang T, Brown JH (2004). Role of ca2+/calmodulin-dependent protein kinase ii in cardiac hypertrophy and heart failure. Cardiovasc Res.

[61] Sumi M, Kiuchi K, Ishikawa T (1991). The newly synthesized selective ca2+/calmodulin dependent protein kinase ii inhibitor kn-93 reduces dopamine contents in pc12h cells. Biochem Biophys Res Commun.

[62] Anderson ME, Braun AP, Wu Y (1998). Kn-93, an inhibitor of multifunctional ca++/calmodulin-dependent protein kinase, decreases early after depolarizations in rabbit heart. J Pharmacol Exp Ther.

[63] Rezazadeh S, Claydon TW, Fedida D (2006). Kn-93 (2-[n-(2-hydroxyethyl)]-n-(4-methoxybenzenesulfonyl)]amino-n-(4-chlorocinnamyl)-n -methylbenzylamine), a calcium/calmodulin-dependent protein kinase ii inhibitor, is a direct extracellular blocker of voltage-gated potassium channels. J Pharmacol Exp Ther.

[64] Ather S, Wang W, Wang Q (2013). Inhibition of camkii phosphorylation of ryr2 prevents inducible ventricular arrhythmias in mice with duchenne muscular dystrophy. Heart Rhythm.

[65] Bapat A, Nguyen TP, Lee JH (2012). Enhanced sensitivity of aged fibrotic hearts to angiotensin ii- and hypokalemia-induced early after depolarization-mediated ventricular arrhythmias. Am J Physiol Heart Circ Physiol.

[66] Said M, Becerra R, Valverde CA (2011). Calcium-calmodulin dependent protein kinase ii (camkii): a main signal responsible for early reperfusion arrhythmias. J Mol Cell Cardiol.

[67] Morita N, Lee JH, Bapat A (2011). Glycolytic inhibition causes spontaneous ventricular fibrillation in aged hearts. Am J Physiol Heart Circ Physiol.

[68] Liu N, Ruan Y, Denegri M (2011). Calmodulin kinase ii inhibition prevents arrhythmias in ryr2(r4496c+/−) mice with catecholaminergic polymorphic ventricular tachycardia. J Mol Cell Cardiol.

[69] Sag CM, Wadsack DP, Khabbazzadeh S (2009). Calcium/calmodulin-dependent protein kinase ii contributes to cardiac arrhythmogenesis in heart failure. Circ Heart Fail.

[70] Pu J, Zhang CT, Bai R (2005). Calmodulin antagonist inhibits torsade de pointes induced by d-sotalol in an isolated rabbit heart model. Zhonghua Xin Xue Guan Bing Za Zhi.

[71] Kirchhof P, Fabritz L, Kilic A (2004). Ventricular arrhythmias, increased cardiac calmodulin kinase ii expression, and altered repolarization kinetics in anp receptor deficient mice. J Mol Cell Cardiol.

[72] Gbadebo TD, Trimble RW, Khoo MS (2002). Calmodulin inhibitor w-7 unmasks a novel electrocardiographic parameter that predicts initiation of torsade de pointes. Circulation.

[73] Mazur A, Roden DM, Anderson ME (1999). Systemic administration of calmodulin antagonist w-7 or protein kinase a inhibitor h-8 prevents torsade de pointes in rabbits. Circulation.

[74] Hidaka H, Yamaki T, Asano M (1978). Involvement of calcium in cyclic nucleotide metabolism in human vascular smooth muscle. Blood Vessels.

[75] Undrovinas AI, Fleidervish IA, Makielski JC (1992). Inward sodium current at resting potentials in single cardiac myocytes induced by the ischemic metabolite lysophosphatidylcholine. Circ Res.

[76] Antzelevitch C, Belardinelli L, Zygmunt AC (2004). Electrophysiological effects of ranolazine, a novel antianginal agent with antiarrhythmic properties. Circulation.

[77] Morita N, Lee JH, Xie Y (2011). Suppression of re-entrant and multifocal ventricular fibrillation by the late sodium current blocker ranolazine. J Am Coll Cardiol.

[78] Sossalla S, Maurer U, Schotola H (2011). Diastolic dysfunction and arrhythmias caused by overexpression of camkiidelta(c) can be reversed by inhibition of late na(+) current. Basic Res Cardiol.

[79] Antoons G, Oros A, Beekman JD (2010). Late na(+) current inhibition by ranolazine reduces torsades de pointes in the chronic atrioventricular block dog model. J Am Coll Cardiol.

[80] Dhalla AK, Wang WQ, Dow J (2009). Ranolazine, an antianginal agent, markedly reduces ventricular arrhythmias induced by ischemia and ischemia-reperfusion. Am J Physiol Heart Circ Physiol.

[81] Scirica BM, Morrow DA, Hod H (2007). Effect of ranolazine, an antianginal agent with novel electrophysiological properties, on the incidence of arrhythmias in patients with non st-segment elevation acute coronary syndrome: results from the metabolic efficiency with ranolazine for less ischemia in non st-elevation acute coronary syndrome thrombolysis in myocardial infarction 36 (merlin-timi 36) randomized controlled trial. Circulation.

[82] Song Y, Shryock JC, Wagner S (2006). Blocking late sodium current reduces hydrogen peroxide-induced arrhythmogenic activity and contractile dysfunction. J Pharmacol Exp Ther.

[83] Undrovinas AI, Belardinelli L, Undrovinas NA (2006). Ranolazine improves abnormal repolarization and contraction in left ventricular myocytes of dogs with heart failure by inhibiting late sodium current. J Cardiovasc Electrophysiol.

[84] Wu L, Shryock JC, Song Y (2004). Antiarrhythmic effects of ranolazine in a guinea pig in vitro model of long-qt syndrome. J Pharmacol Exp Ther.

[85] Fraser H, Belardinelli L, Wang L (2006). Ranolazine decreases diastolic calcium accumulation caused by atx-ii or ischemia in rat hearts. J Mol Cell Cardiol.

[86] Song Y, Shryock JC, Wu L (2004). Antagonism by ranolazine of the pro-arrhythmic effects of increasing late ina in guinea pig ventricular myocytes. J Cardiovasc Pharmacol.

[87] Sicouri S, Belardinelli L, Antzelevitch C (2013). Antiarrhythmic effects of the highly selective late sodium channel current blocker gs-458967. Heart Rhythm.

[88] Belardinelli L, Liu G, Smith-Maxwell C (2013). A novel, potent, and selective inhibitor of cardiac late sodium current suppresses experimental arrhythmias. J Pharmacol Exp Ther.

[89] Yang ZF, Li CZ, Wang W (2011). Electrophysiological mechanisms of sophocarpine as a potential antiarrhythmic agent. Acta Pharmacol Sin.

[90] Zhang S, Ma J, Zhang P, Luo A, Ren Z, Kong L. Sophocarpine attenuates the na+-dependent ca2+ overload induced by anemonia sulcata toxin ii-increased late sodium current in rabbit ventricular myocytes. J Cardiovasc Pharmacol. 2012;60:357–66. 10.1097/FJC.0b013e318262c932 23064241

[91] Ke J, Chen F, Zhang C (2012). Effects of calmodulin-dependent protein kinase ii inhibitor, kn-93, on electrophysiological features of rabbit hypertrophic cardiac myocytes. J Huazhong Univ Sci Technol Med Sci.

[92] Fukunaga K, Muller D, Miyamoto E (1996). Cam kinase ii in long-term potentiation. Neurochem Int.

[93] Ashpole NM, Song W, Brustovetsky T (2012). Calcium/calmodulin-dependent protein kinase ii (camkii) inhibition induces neurotoxicity via dysregulation of glutamate/calcium signaling and hyperexcitability. J Biol Chem.

